# Interplay between *ESR1*/*PIK3CA* codon variants, oncogenic pathway alterations and clinical phenotype in patients with metastatic breast cancer (MBC): comprehensive circulating tumor DNA (ctDNA) analysis

**DOI:** 10.1186/s13058-023-01718-0

**Published:** 2023-10-02

**Authors:** Lorenzo Gerratana, Andrew A. Davis, Marko Velimirovic, Katherine Clifton, Whitney L. Hensing, Ami N. Shah, Charles S. Dai, Carolina Reduzzi, Paolo D’Amico, Firas Wehbe, Arielle Medford, Seth A. Wander, William J. Gradishar, Amir Behdad, Fabio Puglisi, Cynthia X. Ma, Aditya Bardia, Massimo Cristofanilli

**Affiliations:** 1 Department of Medical Oncology, CRO Aviano, National Cancer Institute, IRCCS, Aviano, Italy; 2 Division of Oncology, Department of Medicine, Washington University School of Medicine, St. Louis, MO USA; 3 Massachusetts General Hospital, Boston, MA USA; 4 Harvard Medical School, Boston, MA USA; 5 Feinberg School of Medicine, Northwestern University, Chicago, IL USA; 6 Weill Cornell Medicine, 420 E 70th St, LH 204, New York, NY 10021 USA; 7 Department of Medicine, University of Udine, Udine, Italy

**Keywords:** Circulating biomarker, Endocrine therapy, Next generation sequencing

## Abstract

**Background:**

although being central for the biology and druggability of hormone-receptor positive, HER2 negative metastatic breast cancer (MBC), ESR1 and PIK3CA mutations are simplistically dichotomized as mutated or wild type in current clinical practice.

**Methods:**

The study analyzed a multi-institutional cohort comprising 703 patients with luminal-like MBC characterized for circulating tumor DNA through next generation sequencing (NGS). Pathway classification was defined based on previous work (i.e., RTK, RAS, RAF, MEK, NRF2, ER, WNT, MYC, P53, cell cycle, notch, PI3K). Single nucleotide variations (SNVs) were annotated for their oncogenicity through OncoKB. Only pathogenic variants were included in the models. Associations among clinical characteristics, pathway classification, and *ESR1*/*PIK3CA* codon variants were explored.

**Results:**

The results showed a differential pattern of associations for ESR1 and PIK3CA codon variants in terms of co-occurring pathway alterations patterns of metastatic dissemination, and prognosis. ESR1 537 was associated with SNVs in the ER and RAF pathways, CNVs in the MYC pathway and bone metastases, while ESR1 538 with SNVs in the cell cycle pathway and liver metastases. PIK3CA 1047 and 542 were associated with CNVs in the PI3K pathway and with bone metastases.

**Conclusions:**

The study demonstrated how *ESR1* and *PIK3CA* codon variants, together with alterations in specific oncogenic pathways, can differentially impact the biology and clinical phenotype of luminal-like MBC. As novel endocrine therapy agents such as selective estrogen receptor degraders (SERDS) and PI3K inhibitors are being developed, these results highlight the pivotal role of ctDNA NGS to describe tumor evolution and optimize clinical decision making.

**Supplementary Information:**

The online version contains supplementary material available at 10.1186/s13058-023-01718-0.

## Background

Metastatic breast cancer (MBC) is a treatable yet virtually incurable disease, and most deaths from breast cancer occur due to metastasis. [1, 2]. MBC often evolves via acquisition of new resistant mutations usually under the pressure of anticancer treatments. *ESR1* and *PIK3CA* mutations have been the most thoroughly studied and have been implicated in the biology and druggability of hormone-receptor positive (HR +), HER2-negative (HER2−) MBC, and have important implications for therapeutic selection [3, 4].

Alterations in the activity and expression of estrogen receptor α (ER) are often involved in MBC resistance and progression. Resistance may occur through loss of ER expression, increased expression of ER or related cofactors, post-translational modifications of ER, and/or delocalization of ER to the cellular membrane [5]. Alterations of genes involved in other pathways or epigenetic alterations of *ESR1* promoters can deregulate expression due to pathway crosstalk and modified ER activity [6–8]. Point mutations are the most common *ESR1* genetic alterations and generally arise in the ligand binding domain, most commonly in codons 538, 537, 380, and 536 [5, 8, 9]. In some situations, gene amplifications, deletions, or translocations resulting in fusions can occur [10–12].

*PIK3CA* mutations can occur both as single and multiple concomitant mutations. The frequency of multiple co-occurring *PIK3CA* mutations has been estimated to be approximately 8–13% with the vast majority being double mutations (88–96% among patients with multiple PIK3CA mutations) [13]. Double–*PIK3CA*-mutant breast cancer generally consists of a combination of a *major-mutant* hotspot (either E542, E545, or H1047) and a *minor-mutant* site (either E453, E726, or M1043) [13]

Liquid biopsies that include circulating tumor DNA (ctDNA), circulating tumor cells (CTCs) and exosomes are non-invasive diagnostic tools that are being explored in real-time early cancer detection, monitoring for minimal residual disease, and longitudinal tracking of clonal evolution in the peripheral blood [14, 15]. Due to their increasing sensitivity and decreasing cost, high throughput genomic technologies such as next-generation sequencing (NGS) are becoming widely available [16, 17]. Together with its longitudinal application and its increasing deployment in clinic, ctDNA has become a promising tool for the development of further insight related to MBC’s biological evolution [18–20].

The aim of this retrospective study was to evaluate the interplay between oncogenic pathway alterations and *ESR1*/*PIK3CA* codon variants as these findings relate to their impact on the biological and clinical behavior of HR + HER2− MBC.

## Materials and methods

### Study population and design

This study retrospectively analyzed a multi-institutional cohort of 703 HR + HER2− MBC patients with ctDNA NGS sampling before starting a new treatment. Samples were collected from patients who underwent standard-of-care ctDNA testing at Northwestern University (Chicago, IL), Massachusetts General Hospital (Boston, MA) and Washington University in St. Louis (St. Louis, MO) between 2015 and 2020. No selection was made based on current or prior lines of therapy.

Baseline imaging was performed prior to ctDNA collection and start of therapy according to the treating physician’s choice [e.g., Computed Tomography (CT), Positron Emission Tomography (PET)]. Sites of metastasis were categorized based on the presence of specific organ involvement (e.g., liver involvement, yes vs. no) independently from other metastatic sites.

### ctDNA sample collection and analysis

Two 10-mL samples of whole blood were collected for each patient using standard stabilizing tubes (Streck, NE) at baseline before treatment start. Samples were analyzed using the commercial Guardant360™ NGS platform (Guardant Health, CA), a 72-gene panel based on single-molecule digital sequencing was utilized to detect somatic single nucleotide variants (SNVs), insertions/deletions (indels), gene fusions/rearrangements and copy number variations (CNVs) [21–23]. Mutations were annotated through the OncoKB database according to their effect (loss of function, gain of function) and pathogenicity [24]. Only pathogenic mutations based on OncoKB were included in the logistic and Cox regression models.

Pathway classification was based on previously defined profiles generated on the Cancer Genome Atlas database (i.e., RTK, RAS, RAF, MEK, NRF2, ER, WNT, MYC, P53, cell cycle, Notch, PI3K) [25]. *ESR1* and *PIK3CA* SNVs were analyzed at a codon variant level.

Mutant allele frequency was analyzed both for each *ESR1* and *PIK3CA* codon variant (codon MAF) and based on the highest frequency across all mutated gene detected in the patient’s blood sample (Overall MAF). CNVs were dichotically considered as present/absent.

### Statistical analysis

Clinical and pathologic variables were reported using descriptive analyses. Categorical variables were reported as frequency distributions, whereas continuous variables were described through median and interquartile ranges (IQRs).

Associations between clinical characteristics, pathway classification, and *ESR1*/*PIK3CA* codon variants were explored through uni- and multivariable logistic regression, inclusive of odds ratio (OR) and 95% confidence interval (95% CI) computation.

Overall survival (OS) was defined as the time from the baseline ctDNA blood draw to death from any cause. Patients without an end point event at the last follow-up visit were censored. Differences in survival were tested by log-rank test and uni- and multivariable Cox regression with 95% CI and represented by Kaplan–Meier estimator plot. Correction for significant clinical variables after univariable testing was applied to the multivariable model (i.e., previous treatment with CDK4/6i, number of lines, lung, liver, bone, and soft tissue involvement).

Statistical analysis was conducted using StataCorp 2019 Stata Statistical Software: Release 16.1 (College Station, TX), R (version 4.1.0; The R foundation for Statistical Computing, Vienna, Austria) and JMP (version 16; SAS Institute, Cary, NC).

## Results

### Cohort characteristics and detected gene alterations

The cohort included 703 patients diagnosed with HR + HER2− MBC. In detail, the study included 509 patients (85%) with invasive ductal carcinoma (IDC) and 93 patients (15%) with invasive lobular carcinoma (ILC) (Table 1). The most common metastatic site was bone (548 patients, 78%), followed by liver (290 patients, 41%), lymph nodes (262 patients, 37.3%) and lung (221 patients, 31.5%) (Table 1).

**Table 1:** Clinicopathologic characteristics of the luminal-like MBC cohort.

Characteristics	No	%
BC histological type		
NST	509	84.55
Lobular	93	15.45
BC IHC characteristics		
ER +	697	99.15
PR +	464	66.67
HER2+	0	0
Metastatic involvement		
Bone	548	78.06
Liver	290	41.31
Lymph nodes	262	37.32
Lung	221	31.48
Soft tissue	125	17.81
CNS	46	6.55
Previous treatments		
ET	452	74.10
CT	257	42.13
CDK4/6 inhibitors	322	52.79
mTOR inhibitors	105	17.21
PI3K inhibitors	38	6.23
Immunotherapy	8	1.31

NST (Non-Special Type), CNS (Central Nervous System), ET (Endocrine Therapy), CT (Chemotherapy)

Endocrine therapy (ET) was the most common previous treatment (452 patients, 74%), CDK4/6 inhibitors were the most frequent targeted therapy (322 patients, 52.8%), followed by mTOR inhibitors (105 patients, 17.2%) and PI3K inhibitors (38 patients, 6.2%) (Table 1).

**Fig. 1:**
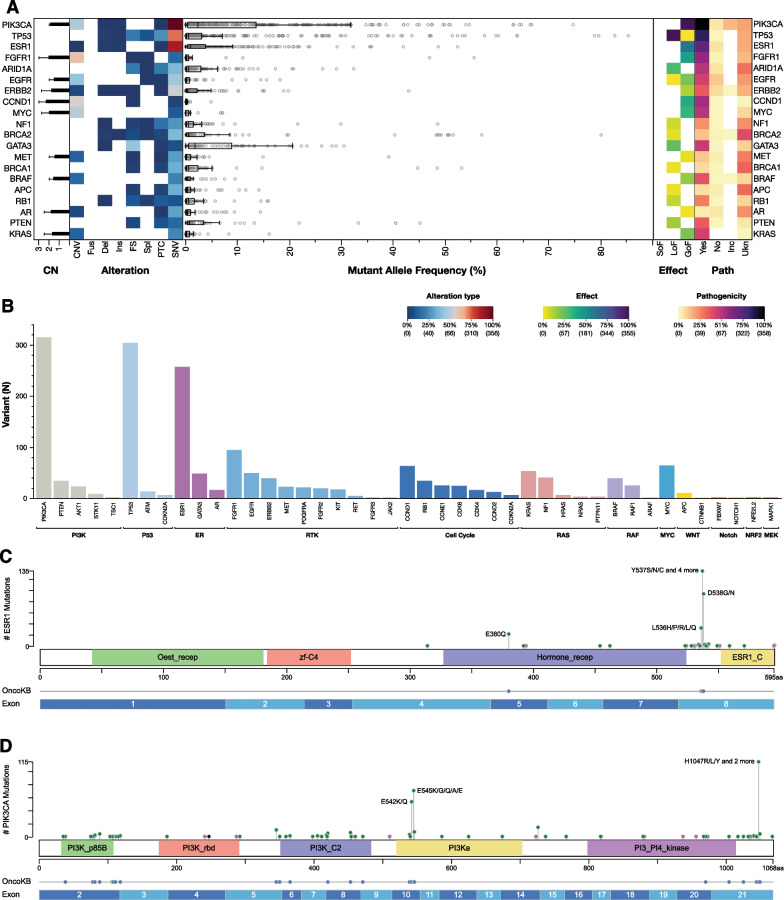
Landscape plot of all detectable aberrations in ctDNA samples. **A** Incidence of the single aberrations [copy number variations (CNV), Fusions (Fus), deletions (Del), insertions (Ins), frameshift (FS), splicing variants (Spl), premature termination codons (PTC) and single nucleotide variation (SNV)] is represented on the left. The mutant allele frequency (MAF) of each mutation is shown in the middle. Effect [gain of function (GOF), loss of function (LOF) and switch of function (SOF)] and pathogenicity [yes, no, unknown (Ukn) and inconclusive (Inc)] of all the detected aberrations are show on the right. Histogram representing different frequency distribution of gene mutations across oncogenic pathways (**B**). Lollipop plot showing the distribution of ESR1 (**C**) and PIK3CA (**D**) mutations by their amino acid coordinates across ER and PI3K domains and across ESR1 and PIK3CA exon sequencies. Oncogenic mutations are highlighted

Across the tested genes, *PIK3CA*, *TP53* and *ESR1* were the most altered. As expected, the most likely effect of the detected SNVs was gain of function (GOF) for *PIK3CA* and *ESR1* and loss of function (LOF) for *TP53* (Fig. 1A, B).

SNVs alterations were mainly observed in the PI3K (35%), P53 (32%), ER (28%), RAS (8%), RTK (7%) and cell cycle (5%) pathways. Copy number variations (CNVs) were mostly detected in the RTK (20%), cell cycle (15%), MYC (7%) PI3K (8%) and RAF (6%) pathways (Fig. 1B).

*ESR1* mutations were detected in 166 patients (24%) and *PIK3CA* in 214 patients (30.5%) (Additional file 1: Table S1). The most common *ESR1* pathogenic mutations among *ESR1*-mutated patients were found in codons 537 (52 patients, 31%), 538 (34 patients, 20%), 536 (14 patients, 8%) and 380 (12 patients, 7%) (Fig. 1C, Additional file 1: Table S1). The most common *PIK3CA* codon variants were 1047 (68 patients, 32%), 545 (47 patients, 22%), and 542 (38 patients, 18%) (Fig. 1D, Additional file 1: Table S1).

Other pathogenic *PIK3CA* SNVs were observed in 28.5% (N = 61) of patients, 16.82% (N = 36) had more than one *PIK3CA* SNV. Among patients with *ESR1* mutations, 33% (N = 54) had polyclonal alterations (Additional file 1: Table S1).

The top 10 genes were CNVs were detected were *FGFR1* (12.1%), *MYC* (9.1%), *CCND1* (8.8%), *PIK3CA* (8.3%), *EGFR* (6.8%), *BRAF* (3.8%), *CDK6* (3.4%), *RAF1* (3.3%), *CCNE1* (3.3%) and *KRAS* (3.1%) (Additional file 1: Table S2).

### *ESR1* and *PIK3CA* codon variants are associated with different ctDNA alterations across oncogenic pathways

**Fig. 2:**
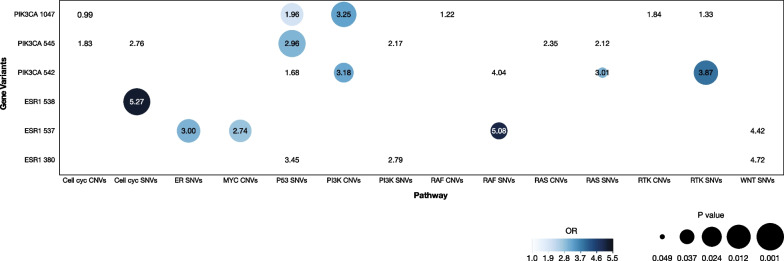
Heat-map showing the association of the main ESR1 and PIK3CA codon variants with concomitant ctDNA alterations in other oncogenic pathways. *PIK3CA* 1047 was associated with SNVs in the P53 pathway and CNVs in the PI3K, while *PIK3CA* 542 with CNVs in the PI3K pathway, SNVs in the RTK and SNVs in the RAS and *PIK3CA* 545 with SNVs in the P53 pathway. *ESR1* 537 was associated with SNVs in the ER pathway, CNVs in the MYC and SNVs in the RAF, *ESR1* 538 with SNVs in cell cycle pathway. Color intensity was proportional to Odds Ratio, while P value was described by size (the lower the P value, the bigger the circle). Syn, Synonymous; Ukn, Unknown; CNVs, Copy Number Variations; SNVs, Single Nucleotide Variations

Specific codon MAF was tested across *PIK3CA* and *ESR1* variants (Fig. 2). No significant differences were observed for *ESR1* (Additional file 1: Fig. S1A). *PIK3CA* codon variants, in contrast, showed a statistically significant difference (P < 0.0001) (Additional file 1: Fig. S1B) with *PIK3CA* 1047 and 542 showing the highest codon MAF (Additional file 1: Fig. S1B).

The association between the main *ESR1* and *PIK3CA* codon variants (Fig. 1C, D) and concomitant gene alterations was investigated according to oncogenic pathway classification.

After multivariable logistic regression, *PIK3CA* 1047 was significantly associated with SNVs in the P53 pathway and CNVs in the PI3K (respectively OR 1.96, 95%CI 1.13–3.39 P = 0.016 and OR 3.25, 95%CI 1.36–7.75 P = 0.008) (Fig. 2, Additional file 1: Table S3).

*PIK3CA* 542 was significantly associated with CNVs in the PI3K pathway, SNVs in the RTK and the RAS pathways (respectively OR 3.18, 95%CI 1.15–8.76 P = 0.025 and OR 3.87, 95%CI 1.36–10.98 P = 0.011, OR 3.01, 95%CI 1.03–8.77 P = 0.044) (Fig. 2, Additional file 1: Table S3).

*PIK3CA* 545 was significantly associated with SNVs in the P53 pathway (OR 2.96, 95%CI 1.54–5.66 P = 0.001) (Fig. 2, Additional file 1: Table S3).

*ESR1* 537 was significantly associated with SNVs in the ER pathway, CNVs in the MYC pathway and SNVs in the RAF pathway (respectively OR 3.00, 95%CI 1.25–7.17 P = 0.014 and OR 2.74, 95%CI 1.20–6.23 P = 0.017, OR 5.08, 95%CI 1.15–22.40 P = 0.032) (Fig. 2, Additional file 1: Table S3).

*ESR1* 538 was significantly associated with SNVs in the cell cycle pathway (OR 5.27, 95%CI 1.82–15.30 P = 0.002) (Fig. 2, Additional file 1: Table S3).

No concomitant alterations were confirmed after multivariable analysis for *ESR1* 380, although a numerical difference was highlighted for SNVs in the P53 pathway (Fig. 2, Additional file 1: Table S3).

No associations were observed for *ESR1* 536.

### Alterations in oncogenic pathways and *ESR1* / *PIK3CA* codon variants are differentially associated with sites of metastasis

The association between *ESR1*/*PIK3CA* codon variants and alterations in oncogenic pathways were then investigated across the main metastatic sites. Correction for number of lines was applied.

**Fig. 3:**
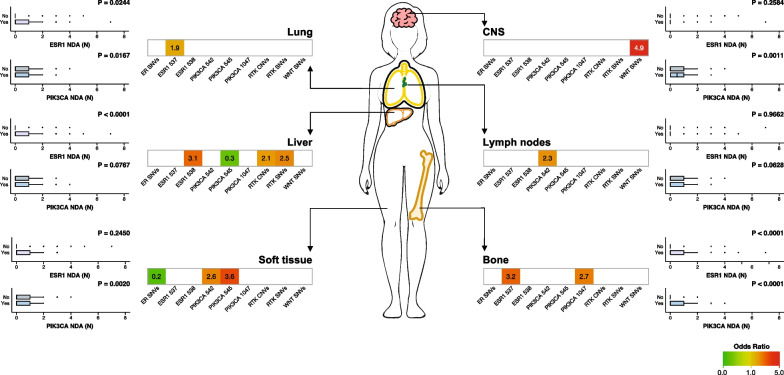
Association of ESR1 and PIK3CA codon variants and alterations in oncogenic pathways with different involvement of the main metastatic sites. (lung, liver, soft tissue, CNS, lymph nodes and bone). The number of concomitant detected aberrations (NDA) of ESR1 and PIK3CA across different metastatic sites was represented next to each metastatic site

After multivariable logistic regression, *ESR1* 537 alterations were significantly associated with bone and lung involvement (respectively OR 3.15, 95%CI 1.08–9.23, P = 0.036, OR 1.89, 95%CI 1.01–3.52, P = 0.046), while *ESR1* 538 alterations were associated with liver metastases only (OR 3.06, 95%CI 1.29–7.29 P = 0.012) (Fig. 3, Additional file 1: Table S4).

*PIK3CA* 1047 alterations were associated with bone metastases (OR 2.68, 95%CI: 1.02–7.05, P = 0.046) (Fig. 3, Additional file 1: Table S4). *PIK3CA* 542 alterations were associated with soft tissue and lymph nodes (respectively OR 2.56, 95%CI: 1.14–5.74, P = 0.022 and OR 2.32, 95% CI 1.15–4.68, P = 0.018) (Fig. 3, Additional file 1: Table S4). *PIK3CA* 545 alterations were associated with soft tissue involvement (OR 3.6, 95%CI 1.76–7.35, P < 0.001) and less associated with liver metastases (OR 0.3, 95%CI 0.13–0.72, P = 0.007) (Fig. 3, Additional file 1: Table S4).

SNVs and CNVs in the RTK pathway were significantly associated with liver metastases (respectively OR 2.47, 95%CI: 1.04–5.85, P = 0.04 and OR 2.12, 95% CI 1.15–3.94, P = 0.017) (Fig. 3, Additional file 1: Table S4). SNVs in the WNT pathway were associated with CNS metastases (OR 4.91, 95% CI 1.17–20.54, P = 0.029) (Fig. 3, Additional file 1: Table S4). SNVs in the ER pathway were less represented in patients with soft tissue involvement (OR 0.2, 95% CI: 0.05–0.86, P = 0.03) (Fig. 3, Additional file 1: Table S4).

The number of concomitant *ESR1* and *PIK3CA* mutations was then analyzed across metastatic sites.

Patients with lung, liver and bone metastases had a significantly higher number of *ESR1* mutations (respectively P = 0.0244, P < 0.0001 and P < 0.0001) (Fig. 3), while *PIK3CA* mutations were significantly higher in patients with lung, CNS, and bone metastases (respectively P = 0.0167, P = 0.0011 and P < 0.0001) and significantly lower in patients with soft tissue involvement (P = 0.0020) (Fig. 3).

### *ESR1* mutations impact on overall survival together with alterations in PI3K, MYC, RAS and P53 pathways

**Fig. 4:**
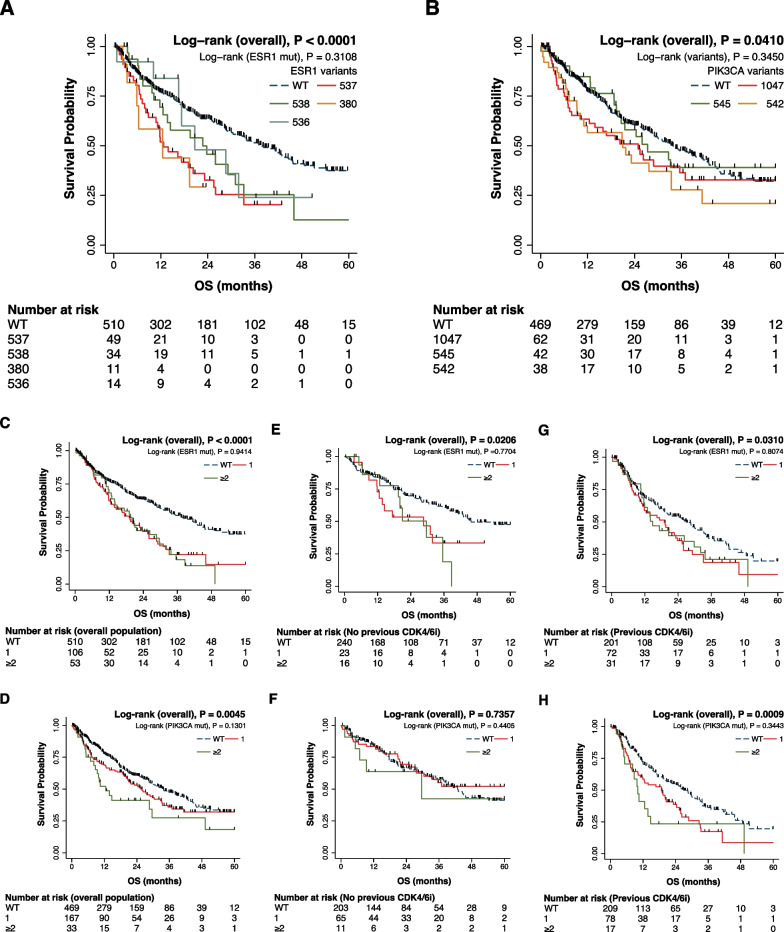
Kaplan–Meier plots for the impact on overall survival (OS) of ESR1 and PIK3CA mutations. The detection of ESR1 or PIK3CA mutations had a significantly unfavorable impact on OS, however, no differential impact was observed across ESR1 or PIK3CA codon variants (**A**, **B**). Similarly, no differential prognostic impact was observed between number of concomitant ESR1 or PIK3CA alterations in the overall population (**C**, **D**) and according to previous CDK4/6i exposure (**E**–**H**). CNVs, Copy Number Variations; SNVs, Single Nucleotide Variations

The prognostic impact of *ESR1* and *PIK3CA* codon variants and number of concomitant mutations was investigated in terms of OS (Fig. 4). Although the detection of *ESR1* or *PIK3CA* mutations had a significantly unfavourable impact on OS (respectively P < 0.0001 and P = 0.0410) (Fig. 4A, B), no differential impact was observed across codon variants (P = 0.3108 and P = 0.3450) (Fig. 4A, B) or the number of concomitant *ESR1* or *PIK3CA* SNVs (P = 0.9414 and P = 0.1301) (Fig. 4C, D). Similar results were observed for patients previously treated with CDK4/6i both for the overall impact of the *ESR1* and *PIK3CA* mutational status (respectively P = 0.0310 and P = 0.0009) and multiple concomitant SNVs (respectively P = 0.8074 and P = 0.3443) (Fig. 4E, F). On the other hand, in patients not previously exposed to CDK4/6i only ESR1 had a significant impact on OS (P = 0.0206) (Fig. 4G, H).

Similar results were observed in terms of PFS in patients treated with endocrine therapy (Additional file 1: Fig. S2A–D), apart from a numerical difference according to number of PIK3CA alterations within patients with PIK3CA-mutated MBC (P = 0.0753) (Additional file 1: Fig. S2D).

After multivariable analysis corrected for previous treatment with CDK4/6i, number of lines, lung, liver, bone, and soft tissue involvement, the prognostic impact of *ESR1* 380 was confirmed (HR 2.48, 95%CI 1.01–6.07, P < 0.048), together with RAS pathway SNVs (HR 1.96, 95%CI 1.16–3.33, P = 0.013), P53 pathway SNVs (HR 1.38, 95%CI 1.17–3.17, P = 0.034), MYC pathway CNVs (HR 1.91, 95%CI 1.08–3.38, P = 0.027), and PI3K CNVs (HR 2.27, 95%CI 1.16–4.47, P = 0.017) (Table 2). Number of lines, liver, bone and soft tissue involvement had an independent impact in terms of OS (Table 2).

**Table 2:** Association of ESR1 and PIK3CA codon variants and alterations in oncogenic pathways with overall survival (OS).

	HR	95% C.I		P value
ESR1 variants				
Wild type	1			
537	1.08	0.68	1.71	0.7594
538	0.77	0.46	1.32	0.3446
380	2.48	1.01	6.07	0.0476
536	1.06	0.41	2.74	0.9071
PIK3CA variants				
Wild type	1			
1047	0.97	0.6	1.55	0.8871
545	0.61	0.34	1.08	0.089
542	1.12	0.65	1.91	0.6849
RAS pathway SNVs				
Not altered	1			
Altered	1.96	1.16	3.33	0.0126
P53 pathway SNVs				
Not altered	1			
Altered	1.38	1.02	1.86	0.0341
Cell cycle pathway SNVs				
Not altered	1			
Altered	1.07	0.53	2.16	0.8576
ER pathway SNVs				
Not altered	1			
Altered	1.41	0.84	2.36	0.1948
PI3K pathway SNVs				
Not altered	1			
Altered	1.57	0.93	2.66	0.0925
RTK pathway CNVs				
Not altered	1			
Altered	1.2	0.77	1.86	0.4269
RAS pathway CNVs				
Not altered	1			
Altered	0.84	0.34	2.08	0.7009
RAF pathway CNVs				
Not altered	1			
Altered	0.91	0.32	2.55	0.8563
ER pathway CNVs				
Not altered	1			
Altered	0.93	0.23	3.83	0.9222
MYC pathway CNVs				
Not altered	1			
Altered	1.91	1.08	3.38	0.027
Cell cycle pathway CNVs				
Not altered	1			
Altered	1.27	0.79	2.05	0.3275
PI3K pathway CNVs				
Not altered	1			
Altered	2.27	1.16	4.47	0.0174
Lung involvement				
No	1			
Yes	1.28	0.96	1.73	0.0961
Liver involvement				
No	1			
Yes	1.84	1.36	2.5	0.0001
Bone involvement				
No	1			
Yes	1.66	1.13	2.43	0.0093
Soft tissue involvement				
No	1			
Yes	2.01	1.41	2.85	0.0001
Previous CDK4/6i				
No	1			
Yes	1.14	0.82	1.58	0.4464
Treatment line				
First	1			
Second	2.24	1.39	3.63	0.001
Third	3.58	2.16	5.94	< 0.0001
Fourth	2.44	1.34	4.43	0.0035
Fifth and beyond	3.52	2.18	5.68	< 0.0001

CNVs, Copy Number Variations; SNVs, Single Nucleotide Variations

## Discussion

Although pivotal in breast cancer biology and evolution, *ESR1* and *PIK3CA* mutations clinically are simplistically dichotomized as mutated or wild type. On the other hand, preclinical data suggests that a more precise characterization of *ESR1* and *PIK3CA* mutated variants may help improve our understanding of how these alterations and associated co-mutations drive resistance and clonal evolution [13, 26–28]. In this study we retrospectively analyzed ctDNA samples from a cohort of 703 patients to more specifically investigate the role of *ESR1* and *PIK3CA* codon variants and other oncogenic pathway alterations and their impact on the clinical phenotype of HR + HER2− MBC.

Similar to previous studies, alterations in *ESR1* and *PIK3CA* were commonly observed in our cohort [29–32]. We investigated differences in clinical phenotypes based on specific codon variants for each of these genes, together with other gene alterations on a pathway level. This approach enabled us to detect potential associations from a biological standpoint that would have been otherwise diluted by the low incidence of the single gene alterations within different pathways. Our results showed a differential pattern of associations for *ESR1* and *PIK3CA* codon variants in terms of co-occurring pathway alterations patterns of metastatic dissemination, and prognosis.

The association among *ESR1*, *PIK3CA* codon variants, and gene alterations was then explored according to oncogenic pathways classification.

*PIK3CA* 1047 and 542 were associated with CNVs in the PI3K pathway. Co-occurring SNVs in the PI3K pathway are well documented and are often associated with exceptional responses. On the other hand, *PIK3CA* CNVs are less well described, since clinical trials focused on PI3K inhibitors are usually focused on PCR-based diagnostic companions and therefore typically report *PIK3CA* mutations but not *PIK3CA* CNVs. Our data suggest the need for a more specific characterization since patients with co-occurring SNVs and CNVs could have different patterns of response [33].

*ESR1* 537 was significantly associated with SNVs in the ER and RAF pathways and CNVs in the MYC pathway, while *ESR1* 538 was associated with SNVs in the cell cycle pathway. The selection of such co-occurring alterations could be the result of prior exposure to ET, suggesting that endocrine resistance may be an emerging property of cellular-wide genetic, epigenetic, and transcriptional phenomena, rather than the result of a single hit aberration [26, 34]. Multiparametric characterizations are therefore needed to better describe this phenomenon and select new therapeutic strategies that could target alterations on a pathway level [35].

It has been previously suggested that gene alterations and expression can influence the development of metastases in different sites [14, 36, 37]. Therefore, in our current study, we combined oncogenic pathways classification and codon variants to better describe how these alterations may impact site of metastasis (e.g., organotropism). Similarly to previous works, *ESR1* mutations were found to be associated with liver and bone metastases and *PIK3CA* with bone. On the other hand, when considered on a codon basis, only *ESR1* 538 was associated with liver metastases, while *ESR1* 537 and *PIK3CA* 1047 were significantly associated with bone metastases. A more complex role of *ESR1* in the biology of HR + HER2− MBC has been suggested by previous data, not only on a genetic but also on an epigenetic standpoint where liver metastases were associated with low methylation levels of the *ESR1* promoter [8, 38–41]. In addition to specific codon variants, we showed that alterations in the RTK pathway were associated with liver metastases, supporting the importance of this pathway in driving therapeutic resistance. Prior studies have shown that alterations in *ESR1*, *AKT1*, *ERBB2*, *FGFR4* and *NF1* are linked to the RTK-RAS axis in patients who develop liver metastases [38]. On one hand therapeutic targeting of these alterations could have implications for future treatment strategies, potentially impacting specific sites of metastatic spread [42]. On the other hand, ctDNA shedding is influenced by tumor burden and may vary across metastatic sites, introducing a potential bias in the interpretation of data linked to organotropism [43].

As expected, our study confirmed the prognostic impact of alterations in *ESR1* and *PIK3CA* on OS in univariable models [44]. This significant impact on prognosis was consistent across codon variants and also in patients with polyclonal *ESR1* or *PIK3CA* mutations (Fig. 4) [13].

As new treatment options specifically targeting ER are gaining momentum, the prognostic and predictive role of *ESR1* mutations codon variants may become central in a significant number of patients with MBC [45]. In terms of oncogenic pathways, our multivariable models highlighted a significant prognostic role for RAS pathway SNVs, MYC pathway CNVs, PI3K pathway CNVs, and P53 pathway SNVs, while only *ESR1* 380 codon variant retained its prognostic impact. The prognostic impact of gene alterations according to oncogenic pathways is an emerging paradigm that has been previously explored in the translational analysis of MONALEESA 7 [20]. RTK gene alterations (defined as either SNVs and CNV in the *FGFR1*, *ERBB2*, *IGF1R*, *EGFR*, *ERBB3*, *KDR*, *KIT*, *PDGFRB*, *PDGFRA*, *ERBB4*, *VEGFA* and *IGF1* genes) were identified in 17% of patients, and similarly to or study, these patients experienced shorter PFS, especially in the ET-only arm [20]. Of note, the incidence of RTK alterations was lower in MONALEESA 7 with respect to our cohort (24%), possibly due to less exposure to prior treatments [20].

Our study has several potential limitations. First, the NGS technology used in our study was not able to define whether multiple detected mutations where concomitantly harbored by the same cell population or if they originated from different subclones. This distinction may play an important role as cis-*PIK3CA* codon variants (i.e., present on the same allele) have been observed to significantly impact treatment response and MBC biology [13]. Similarly, this limitation applies to associations between specific alterations and metastatic organotropism. If on one hand the real-world design of the study may have increased its clinical transferability, on the other it may have added potential bias due to not uniform treatment strategies across institutions, historical cohorts, and treatment lines. Moreover, timing from ctDNA and treatment start was decided on a clinical basis and not per protocol, introducing variability across patients. Finally, current clinical NGS platforms rely on targeted gene panels, potentially underestimating the tumor’s mutational load and leaving out other less represented key genes across the considered oncogenic pathways.

## Conclusions

ESR1 and PIK3CA codon variants, together with alterations in specific oncogenic pathways, can differentially impact the biology, and survival of patients with HR + HER2-negative metastatic breast cancer. As novel therapies such as selective estrogen receptor degraders and PI3K inhibitors are being developed and are entering clinical practice, our results highlight the pivotal role of ctDNA NGS in describing tumor evolution under treatment pressure and optimizing both clinical decision making and future drug development.

### Supplementary Information

**Additional file 1. **Supplementary Tables and Figures.

## Data Availability

The datasets generated and analyzed during the current study are not publicly available due to data protection rules but are available from the corresponding author on reasonable request.

## Abbreviations

95% CI: 95% Confidence interval
CNVs: Copy number variations
CT: Computed Tomography
CTCs: Circulating tumor cells
ctDNA: Circulating tumor DNA
ET: Endocrine therapy
GOF: Gain of function
HER2−: HER2-negative
HR: Hazard ratio
HR +: Hormone-receptor positive
IDC: Invasive ductal carcinoma
ILC: Invasive lobular carcinoma
IQRs: Interquartile ranges
LOF: Loss of function
MAF: Mutant allele frequency
MBC: Metastatic breast cancer
NGS: Next generation sequencing
OR: Odds ratio
OS: Overall survival
PET: Positron Emission Tomography
SERDS: Selective estrogen receptor degraders
SNVs: Single nucleotide variations
