# Supplementary material for: Interplay between ESR1/PIK3CA codon variants, oncogenic pathway alterations and clinical phenotype in patients with metastatic breast cancer (MBC): comprehensive circulating tumor DNA (ctDNA) analysis
Source: Breast Cancer Res. 2023 Oct 2;25:112. doi: 10.1186/s13058-023-01718-0 (PMC10546685; doi:10.1186/s13058-023-01718-0)
Supplement: Supplementary file 1 — Additional file 1. Supplementary Tables and Figures. [file 13058_2023_1718_MOESM1_ESM.docx]

|  | **WT** | **Mut** | | |
| --- | --- | --- | --- | --- |
|  |  | N | % Overall | % Among mutated |
| **ESR1** |  |  | | |
| WT | 537 (76.4%) | 166 (23.6%) | | |
| 537 |  | 52 | 7.4 | 31.3 |
| 538 |  | 34 | 4.8 | 20.5 |
| 536 |  | 14 | 2.0 | 8.4 |
| 380 |  | 12 | 1.7 | 7.2 |
| Polyclonal |  | 54 | 7.7 | 32.5 |
| **PIK3CA** |  |  |  |  |
| WT | 489 (69.6%) | 214 (30.4%) | | |
| 1047 |  | 68 | 9.7 | 31.8 |
| 545 |  | 47 | 6.7 | 22.0 |
| 542 |  | 38 | 5.4 | 17.8 |
| Other/Polyclonal |  | 61 | 8.7 | 28.5 |

**Supplementary table 1**. ESR1 and PIK3CA wt vs mutated gene frequency and ESR1 and PIK3CA codon variant frequency among overall study population and respectively among ESR1 or PIK3CA mutated patients.

|  | **Not amplified** | | **Amplified** | |
| --- | --- | --- | --- | --- |
|  | **N** | **%** | **N** | **%** |
| *FGFR1* | 618 | 87.91 | 85 | 12.09 |
| *MYC* | 639 | 90.9 | 64 | 9.1 |
| *CCND1* | 641 | 91.18 | 62 | 8.82 |
| *PIK3CA* | 645 | 91.75 | 58 | 8.25 |
| *EGFR* | 655 | 93.17 | 48 | 6.83 |
| *BRAF* | 676 | 96.16 | 27 | 3.84 |
| *CDK6* | 679 | 96.59 | 24 | 3.41 |
| *RAF1* | 680 | 96.73 | 23 | 3.27 |
| *CCNE1* | 680 | 96.73 | 23 | 3.27 |
| *KRAS* | 681 | 96.87 | 22 | 3.13 |
| *PDGFRA* | 683 | 97.16 | 20 | 2.84 |
| *MET* | 684 | 97.3 | 19 | 2.7 |
| *CDK4* | 687 | 97.72 | 16 | 2.28 |
| *KIT* | 691 | 98.29 | 12 | 1.71 |
| *CCND2* | 692 | 98.44 | 11 | 1.56 |
| *AR* | 694 | 98.72 | 9 | 1.28 |
| *ERBB2* | 695 | 98.86 | 8 | 1.14 |
| *FGFR2* | 695 | 98.86 | 8 | 1.14 |

**Supplementary table 2**. Prevalence of CNVs across the analyzed population

|  |  | **OR** | **95% C.I.** | | **P value** |
| --- | --- | --- | --- | --- | --- |
| **PIK3CA 542** |  |  |  |  |  |
| PI3K CNVs | not present | 1 |  |  |  |
|  | present | 3.18 | 1.15 | 8.76 | 0.025 |
| P53 SNVs | not present | 1 |  |  |  |
|  | present | 1.68 | 0.81 | 3.49 | 0.166 |
| RAF SNVs | not present | 1 |  |  |  |
|  | present | 4.04 | 0.68 | 24.09 | 0.125 |
| RAS SNVs | not present | 1 |  |  |  |
|  | present | 3.01 | 1.03 | 8.77 | 0.044 |
| RTK SNVs | not present | 1 |  |  |  |
|  | present | 3.87 | 1.36 | 10.98 | 0.011 |
| **PIK3CA 545** |  |  |  |  |  |
| PI3K SNVs | not present | 1 |  |  |  |
|  | present | 2.17 | 0.86 | 5.48 | 0.102 |
| cell cycle CNVs | not present | 1 |  |  |  |
|  | present | 1.83 | 0.83 | 4.05 | 0.136 |
| RAS CNVs | not present | 1 |  |  |  |
|  | present | 2.35 | 0.62 | 8.94 | 0.211 |
| cell cycle SNVs | not present | 1 |  |  |  |
|  | present | 2.76 | 0.92 | 8.25 | 0.069 |
| P53 SNVs | not present | 1 |  |  |  |
|  | present | 2.96 | 1.54 | 5.66 | 0.001 |
| RAS SNVs | not present | 1 |  |  |  |
|  | present | 2.12 | 0.77 | 5.82 | 0.145 |
| **PIK3CA 1047** |  |  |  |  |  |
| PI3K CNVs | not present | 1 |  |  |  |
|  | present | 3.25 | 1.36 | 7.75 | 0.008 |
| cell cycle CNVs | not present | 1 |  |  |  |
|  | present | 0.99 | 0.46 | 2.15 | 0.98 |
| RAF CNVs | not present | 1 |  |  |  |
|  | present | 1.22 | 0.44 | 3.42 | 0.703 |
| RTK CNVs | not present | 1 |  |  |  |
|  | present | 1.84 | 0.92 | 3.67 | 0.085 |
| P53 SNVs | not present | 1 |  |  |  |
|  | present | 1.96 | 1.13 | 3.39 | 0.016 |
| RTK SNVs | not present | 1 |  |  |  |
|  | present | 1.33 | 0.47 | 3.72 | 0.591 |
| **ESR1 380** |  |  |  |  |  |
| PI3K SNVs | not present | 1 |  |  |  |
|  | present | 2.79 | 0.78 | 9.91 | 0.113 |
| P53 SNVs | not present | 1 |  |  |  |
|  | present | 3.45 | 0.98 | 12.17 | 0.055 |
| WNT SNVs | not present | 1 |  |  |  |
|  | present | 4.72 | 0.47 | 47.00 | 0.186 |
| **ESR1 537** |  |  |  |  |  |
| ER SNVs | not present | 1 |  |  |  |
|  | present | 3.00 | 1.25 | 7.17 | 0.014 |
| MYC CNVs | not present | 1 |  |  |  |
|  | present | 2.74 | 1.20 | 6.23 | 0.017 |
| WNT SNVs | not present | 1 |  |  |  |
|  | present | 4.42 | 0.90 | 21.71 | 0.068 |
| RAF SNVs | not present | 1 |  |  |  |
|  | present | 5.08 | 1.15 | 22.40 | 0.032 |
| **ESR1 538** |  |  |  |  |  |
| cell cycle SNVs | not present | 1 |  |  |  |
|  | present | 5.27 | 1.82 | 15.30 | 0.002 |

**Supplementary Table 3**: Association of ESR1 and PIK3CA codon variants with different ctDNA alterations across oncogenic pathways. Abbreviations: CNVs, Copy Number Variations; SNVs, Single Nucleotide Variations.

|  |  | **OR** | **95% C.I.** | | **P value** |
| --- | --- | --- | --- | --- | --- |
| **Bone** | | | | | |
| **PI3K SNVs** | not present | 1 |  |  |  |
|  | present | 1.51 | 0.56 | 4.13 | 0.418 |
| **cell cycle CNVs** | not present | 1 |  |  |  |
|  | present | 1.63 | 0.73 | 3.64 | 0.232 |
| **PIK3CA** | wild type | 1 |  |  |  |
|  | 1047 | 2.68 | 1.02 | 7.05 | 0.046 |
|  | 545 | 2.08 | 0.77 | 5.58 | 0.147 |
|  | 542 | 2.3 | 0.77 | 6.86 | 0.134 |
| **ESR1** | wild type | 1 |  |  |  |
|  | 537 | 3.15 | 1.08 | 9.23 | 0.036 |
|  | 538 | 2.01 | 0.67 | 6.03 | 0.215 |
|  | 380 | 0.82 | 0.15 | 4.41 | 0.818 |
|  | 536 | 2.99 | 0.36 | 24.54 | 0.308 |
| **Treatment line** | First | 1 |  |  |  |
|  | Second | 1.1 | 0.61 | 1.98 | 0.746 |
|  | Third | 0.73 | 0.39 | 1.34 | 0.307 |
|  | Fourth | 0.9 | 0.42 | 1.95 | 0.796 |
|  | Fifth and beyond | 1.83 | 0.94 | 3.54 | 0.074 |
| **Liver** | | | | | |
| **PI3K CNVs** | not present | 1 |  |  |  |
|  | present | 1.61 | 0.66 | 3.94 | 0.293 |
| **cell cycle CNVs** | not present | 1 |  |  |  |
|  | present | 1.37 | 0.71 | 2.66 | 0.353 |
| **MYC CNVs** | not present | 1 |  |  |  |
|  | present | 1.64 | 0.71 | 3.82 | 0.247 |
| **RAF CNVs** | not present | 1 |  |  |  |
|  | present | 0.65 | 0.2 | 2.15 | 0.482 |
| **RAS CNVs** | not present | 1 |  |  |  |
|  | present | 1.21 | 0.3 | 4.95 | 0.787 |
| **RTK CNVs** | not present | 1 |  |  |  |
|  | present | 2.12 | 1.15 | 3.94 | 0.017 |
| **RTK SNVs** | not present | 1 |  |  |  |
|  | present | 2.47 | 1.04 | 5.85 | 0.04 |
| **PIK3CA** | wild type | 1 |  |  |  |
|  | 1047 | 1.28 | 0.65 | 2.5 | 0.475 |
|  | 545 | 0.3 | 0.13 | 0.72 | 0.007 |
|  | 542 | 1.67 | 0.74 | 3.75 | 0.214 |
| **ESR1** | wild type | 1 |  |  |  |
|  | 537 | 1.51 | 0.77 | 2.96 | 0.226 |
|  | 538 | 3.06 | 1.29 | 7.29 | 0.012 |
|  | 380 | 2.89 | 0.61 | 13.55 | 0.179 |
|  | 536 | 1.25 | 0.32 | 4.86 | 0.745 |
| **Treatment line** | First | 1 |  |  |  |
|  | Second | 1.61 | 0.9 | 2.87 | 0.107 |
|  | Third | 1.59 | 0.85 | 2.96 | 0.143 |
|  | Fourth | 2.78 | 1.37 | 5.68 | 0.005 |
|  | Fifth and beyond | 5.73 | 3.26 | 10.07 | <0.001 |
| **Lung** | | | | | |
| **RTK CNVs** | not present | 1 |  |  |  |
|  | present | 1.58 | 0.96 | 2.62 | 0.074 |
| **P53 SNVs** | not present | 1 |  |  |  |
|  | present | 1.29 | 0.85 | 1.95 | 0.236 |
| **PIK3CA** | wild type | 1 |  |  |  |
|  | 1047 | 1.58 | 0.85 | 2.91 | 0.147 |
|  | 545 | 1.02 | 0.49 | 2.13 | 0.948 |
|  | 542 | 0.65 | 0.28 | 1.51 | 0.317 |
| **ESR1** | wild type | 1 |  |  |  |
|  | 537 | 1.89 | 1.01 | 3.52 | 0.046 |
|  | 538 | 1.7 | 0.8 | 3.63 | 0.169 |
|  | 380 | 3.27 | 0.75 | 14.3 | 0.115 |
|  | 536 | 0.26 | 0.03 | 2.1 | 0.206 |
| **Treatment line** | First | 1 |  |  |  |
|  | Second | 0.97 | 0.56 | 1.68 | 0.901 |
|  | Third | 1.04 | 0.57 | 1.89 | 0.896 |
|  | Fourth | 1.37 | 0.68 | 2.75 | 0.381 |
|  | Fifth and beyond | 1.17 | 0.69 | 1.98 | 0.568 |
| **Soft tissue** | | | | | |
| **ER SNVs** | not present | 1 |  |  |  |
|  | present | 0.2 | 0.05 | 0.86 | 0.03 |
| **MYC CNVs** | not present | 1 |  |  |  |
|  | present | 1.71 | 0.83 | 3.52 | 0.144 |
| **RAS SNVs** | not present | 1 |  |  |  |
|  | present | 1.46 | 0.63 | 3.37 | 0.373 |
| **PIK3CA** | wild type | 1 |  |  |  |
|  | 1047 | 1.21 | 0.58 | 2.5 | 0.614 |
|  | 545 | 3.6 | 1.76 | 7.35 | <0.001 |
|  | 542 | 2.56 | 1.14 | 5.74 | 0.022 |
| **Treatment line** | First | 1 |  |  |  |
|  | Second | 0.93 | 0.46 | 1.86 | 0.838 |
|  | Third | 1.21 | 0.58 | 2.51 | 0.614 |
|  | Fourth | 0.93 | 0.37 | 2.37 | 0.884 |
|  | Fifth and beyond | 1.81 | 0.98 | 3.33 | 0.056 |
| **Lymph node** | | | | | |
| **PIK3CA** | wild type | 1 |  |  |  |
|  | 1047 | 1.45 | 0.83 | 2.53 | 0.188 |
|  | 545 | 0.99 | 0.51 | 1.93 | 0.98 |
|  | 542 | 2.32 | 1.15 | 4.68 | 0.018 |
| **Treatment line** | First | 1 |  |  |  |
|  | Second | 0.73 | 0.45 | 1.18 | 0.202 |
|  | Third | 0.73 | 0.42 | 1.27 | 0.263 |
|  | Fourth | 0.65 | 0.34 | 1.26 | 0.199 |
|  | Fifth and beyond | 0.98 | 0.62 | 1.56 | 0.948 |
| **CNS** | | | | | |
| **RTK SNVs** | not present | 1 |  |  |  |
|  | present | 1.64 | 0.57 | 4.7 | 0.355 |
| **WNT SNVs** | not present | 1 |  |  |  |
|  | present | 4.91 | 1.17 | 20.54 | 0.029 |
| **P53 SNVs** | not present | 1 |  |  |  |
|  | present | 1.6 | 0.81 | 3.16 | 0.174 |
| **cell cycle SNVs** | not present | 1 |  |  |  |
|  | present | 1.41 | 0.38 | 5.22 | 0.609 |
| **RTK CNVs** | not present | 1 |  |  |  |
|  | present | 1.66 | 0.8 | 3.46 | 0.172 |
| **PI3K SNVs** | not present | 1 |  |  |  |
|  | present | 1.57 | 0.71 | 3.46 | 0.263 |
| **Treatment line** | First | 1 |  |  |  |
|  | Second | 0.33 | 0.09 | 1.24 | 0.1 |
|  | Third | 0.57 | 0.17 | 1.9 | 0.363 |
|  | Fourth | 1.22 | 0.4 | 3.79 | 0.726 |
|  | Fifth and beyond | 1.41 | 0.63 | 3.2 | 0.404 |

**Supplementary Table 4:** Alterations in oncogenic pathways, ESR1 and PIK3CA codon variants and differential association with metastatic sites. Abbreviations: CNVs, Copy Number Variations; SNVs, Single Nucleotide Variations.

**Supplementary Figure 1: Box plot of the codon Mutant Allele Frequency (MAF) of ESR1 (A) and PIK3CA (C), overall MAF of ESR1 (B) and PIK3CA (D) codon variants.** PIK3CA codon variants, showed a statistically significant difference in codon MAF (P < 0.0001), with PIK3CA 1047 and 542 being the codon variants with the highest codon MAF. PIK3CA showed a statistically significant difference also in overall MAF (P = 0.0073) and other pathogenic codon variants, 542, 1047 and 545 represented the codon variants with the highest overall MAF.

**Supplementary Figure 2: Kaplan-Meier plots for the impact on progression free survival (PFS) of ESR1 and PIK3CA codon variants (A, B) and number of concomitant alterations (C, D).**
